# Endovascular management of tracheo-innominate artery fistula: a case report and literature review

**DOI:** 10.1590/1677-5449.003418

**Published:** 2018

**Authors:** Alexandre de Tarso Machado, Manuela Cristina Ribeiro Dias Barroso

**Affiliations:** 1 Faculdade de Ciências Médicas e da Saúde de Juiz de Fora – SUPREMA, Juiz de Fora, MG, Brasil.

**Keywords:** tracheostomy, stent, hemorrhage, endovascular procedures, emergency treatment

## Abstract

Tracheo-innominate artery fistula (TIF) is a rare complication of tracheostomy, with incidence ranging from 0.1 to 1%, but mortality is high in untreated cases. Early signs range from self-limited bleeding to massive hemorrhage with hypovolemic shock. The caliber of the tracheostomy cannula, its position in contact with the tracheal wall, and tracheal cuff pressure can traumatize the mucosa and trigger development of a TIF. We describe the case of a 14-year-old female patient who had been tracheostomized at the age of eight because of head trauma. She later developed subglottic stenosis requiring dilation sessions for six years. During the fifth year of these sessions, she presented repetitive hemoptysis, initially treated by surgery to implant an expanded polytetrafluoroethylene graft. One year later, she had an intense hemorrhage, which was controlled using endovascular techniques followed by definitive surgery, performed electively. The patient was followed up for six months, without complications.

## INTRODUCTION

 Tracheo-innominate artery fistula (TIF) is a rare complication of tracheostomy, with incidence varying from 0.1 to 1%, but with high mortality in untreated cases. 1 Bleeding generally occurs after manipulation of the trachea such as dilatation and routine cannula changes, or even after coughing. Examinations such as bronchoscopy, arteriography, and computed tomography angiography with 3D reconstruction (CT) can be useful for diagnosing TIF. However, in certain situations they may be inconclusive because of obstruction by clots and adjacent structures masking the bleeding. 2

 In the majority of cases, bleeding is controlled with clinical measures such as volume resuscitation, suspension of anticoagulants, and platelet antiaggregants, and transfusion of blood products. A TIF can also be sealed off by positioning the cannula and inflating a cuff. 1 In the most severe cases, the treatment recommended is exclusion of the vessel responsible for the hemorrhage, by conventional surgical or endovascular repair. 3

 This study describes the case of a patient with TIF treated with endovascular techniques to control severe massive hemorrhage, paying particular attention to its clinical presentation, diagnosis, and treatment. 

## CASE DESCRIPTION

 The patient was a 14-year-old female who had a prior history of tracheostomy, performed when in an intensive care unit because of head trauma, at the age of eight. She had been discharged from hospital after a one month stay and, around three months later, developed subglottic tracheal stenosis, which was treated with outpatient endoscopic dilatation sessions over a period of six years. 

 During the fifth year of these dilatation sessions, she suffered repeated episodes of hemoptysis, without significant hemodynamic consequences, initially managed conservatively. However, bronchoscopy and CT revealed a TIF, which was identified as the source of the bleeding. This lesion was repaired by a surgical procedure to ligate the TIF, reconstruction of the brachiocephalic trunk with an expanded polytetrafluoroethylene (PTFE) prosthesis, preserving the carotid and vertebral arteries. 

 After this initial surgical treatment, she progressed well for one year, during which the dilatation sessions were continued, but at the end of this period, hemoptysis recurred. This time, bronchoscopy and CT of the thoraco-cervical region did not reveal the source of the bleeding. 

**Figure 1:**
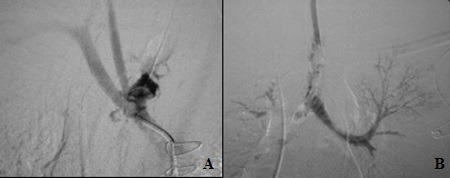
(A) Selective arteriography of the brachiocephalic trunk, identifying the fistula site close to the bifurcation of the subclavian and right common carotid arteries; (B) Contrast in the bronchial tree via selective arteriography of the brachiocephalic trunk.

 Under general anesthesia, selective arteriography of the brachiocephalic trunk revealed a TIF approximately six cm from the carina ( Figure 1 A). At this point, the fistula burst open once more, flooding the lower respiratory tract, with massive bleeding via the oral endotracheal tube, hemomediastinum with compression of the apical segment of the right lung and resultant deterioration of ventilatory function ( Figure 1 B). 

**Figure 2:**
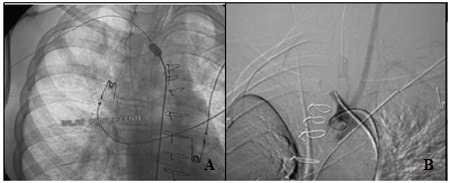
(A) Compliant balloon positioned in a mid-distal segment of the brachiocephalic trunk; (B) Aortography with 5F pigtail catheter via a contralateral femoral access.

 As an emergency measure, a compliant occlusion balloon (Coda®, Cook Medical, Bloomington, United States) was placed in the mid-distal segment of the brachiocephalic trunk, achieving total obstruction of flow through the vessel ( Figures 2 2B). Once the blood had been aspirated through the oral endotracheal tube, the patient’s saturation began to improve and hemodynamic stability was achieved. 

**Figure 3:**
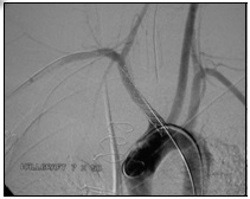
Final control arteriography after placement of a 70 × 50 mm stent (Wallgraft®, Boston Scientific, Marlborough, United States) in the brachiocephalic trunk/right subclavian artery, proximal to the ostium of the right vertebral artery.

 Endovascular treatment of the TIF was conducted with placement of a covered stent measuring 7 × 50 mm (Wallgraft®, Boston Scientific, Marlborough, United States) in the brachiocephalic trunk and right subclavian artery, proximal of the origin of the right vertebral artery, excluding the fistula and occluding the origin of the right common carotid artery, which totally controlled the bleeding ( Figure 3 ). 

 Three days later, an elective surgical operation was conducted via a cervical access to debride devitalized tissues, remove the prostheses and perform carotid-carotid revascularization with preservation of the cervical vessels. One day after the last surgical procedure, the patient was already extubated, breathing spontaneously, hemodynamically stable, and free from signs of neurological deficits. She was put on antibiotics for three weeks and remained free from additional clinical complications over six months of outpatients follow-up. 

## DISCUSSION

 Tracheo-innominate artery fistula is a potentially fatal complication that demands prompt diagnosis and treatment. It may develop after tracheostomy, laryngectomy, or penetrating traumas or may be linked to cancer. In 72% of cases it emerges with three weeks of a tracheostomy, but it can also occur some years after the surgical procedure. 1

 Early signs of development include self-limiting bleeding with spontaneous resolution, known as “sentinel bleeding”, which is present in 35 to 50% of cases, or massive hemorrhage with pulsation of the tracheal cannula. 4 ^,^ 5 Since it is an arterial hemorrhage, bleeding occurs in intermittent, bright red jets of blood and tends to be more severe than a venous hemorrhage, since blood loss is more rapid. This copious bleeding can have sudden onset, with no warning signs. 6

 Trauma to the anterior wall of the trachea, caused by the caliber of the cannula used for tracheostomy, by its being positioned in contact with the tracheal wall, and by pressure exerted by the cuff, may result in an ischemic process, inflammation and ulceration with transmural necrosis of the anterior trachea, involving adjacent structures such as the brachiocephalic trunk. 6 ^,^ 7 Additionally, hyperextension of the neck and poor positioning of the cannula can also cause trauma to mucosa and are thus factors that may trigger development of TIF. 8

 The initial approach in these cases is hyperinflation of the tracheostomy cuff or endotracheal tube, after protection of the airway. If this maneuver fails to staunch the bleeding, digital compression of the anterior tracheal wall should be applied via the opening. 9 The best therapeutic strategy is not to place the tracheostomy tube distal of the fourth tracheal ring; avoid hyperextension of the neck; and maintain cuff pressure below 20 mmHg, to prevent necrosis of the mucosa. 10

 Generally, traumatic arterial lesions are treated using traditional revascularization techniques. However, it is becoming increasingly possible to repair, in an effective way, vascular injuries with minimally invasive procedures. 11 ^,^ 12 The primary advantage of endovascular treatment for TIF is reduction of morbidity and mortality, particularly in more serious cases. 13 Limitations include difficulties with stent placement because of the site of injury, making additional surgical bypasses necessary to maintain blood flow, in addition to a lack of resources in smaller hospitals. Some authors suggest that stents can be used as a temporary treatment to stabilize the patient before a definitive surgical procedure is performed, as was done in this case, whereas others suggest using stents as the definitive treatment. 14

 Even though some TIF cases have been successfully treated with endovascular techniques without a need for supplementary surgery (with embolization, covered stent placement, or temporary occlusion with balloons), the majority of authors are reluctant to use any type of synthetic material in proximity to an infected area. 14 These authors also highly recommend using antibiotics for a prolonged period with patients who have had endovascular treatment. In cases with persistent sepsis or other signs of complications, an open surgical procedure should be considered. 15

 Notwithstanding, covered stents are becoming accepted as an option for treatment of vascular traumas, because placement is a minimally invasive procedure that offers rapid control of bleeding. They can be used as part of a temporary procedure with the objective of avoiding blood loss exsanguination, followed by a definitive treatment, as described here, or treatment may even be definitive in selected cases. 16

## CONCLUSIONS

 In the majority of cases, TIF is managed using conservative measures, without a need for intervention. In certain more serious cases, endovascular treatment can be used as an effective method of bleeding control until definitive reparative surgery can be performed. In this case, the use of a covered stent to control hemorrhage was effective and should be considered in situations with massive bleeding and severe hemodynamic repercussions, particularly in emergencies when the delay involved in a definitive procedure could cost the patient’s life. 
