# Supplementary material for: Endovascular management of tracheo-innominate artery fistula: a case report and literature review
Source: J Vasc Bras. 2018 Oct-Dec;17(4):348–52. doi: 10.1590/1677-5449.003418 (PMC6375266; doi:10.1590/1677-5449.003418)
Supplement: Translation

# Translation: Tratamento endovascular de fístula tronco arterial braquiocefálico-traqueal: relato de caso e revisão de literatura

**Authors:** Alexandre de Tarso Machado, Manuela Cristina Ribeiro Dias Barroso

**Affiliations:** 1 Faculdade de Ciências Médicas e da Saúde de Juiz de Fora – SUPREMA, Juiz de Fora, MG, Brasil.

**Keywords:** traqueostomia, stent, sangramento, procedimentos endovasculares, tratamento de emergência

## Abstract

A fístula do tronco arterial braquiocefálico-traqueal é uma complicação pouco frequente da traqueostomia, com incidência entre 0,1 e 1%, porém com alta mortalidade nos casos não tratados. Sinais precoces incluem desde sangramento autolimitado a hemorragia maciça com choque hipovolêmico. A espessura da cânula de traqueostomia, seu posicionamento junto à parede traqueal e a pressão do balonete traqueal podem lesionar a mucosa e precipitar seu desenvolvimento. Descrevemos o caso de paciente do sexo feminino, 14 anos, traqueostomizada aos oito anos após traumatismo craniencefálico. Evoluiu após esse período com estenose subglótica e necessidade de sessões de dilatação por seis anos. No quinto ano das sessões, apresentou hemoptise de repetição, inicialmente tratada por cirurgia com prótese de politetraflouroetileno expandido. Um ano depois, apresentou novo sangramento com repercussão hemodinâmica, controlada pela técnica endovascular, e nova cirurgia foi programada em caráter eletivo. Após a segunda cirurgia, houve seis meses de acompanhamento clínico ambulatorial, sem novas complicações.

## INTRODUÇÃO

 A fístula do tronco arterial braquiocefálico-traqueal (FTABCT) é uma complicação pouco frequente da traqueostomia, com incidência variando entre 0,1 e 1%, porém com alta mortalidade nos casos não tratados 1 . O sangramento ocorre geralmente após manipulação da traqueia através de dilatação, troca habitual da cânula e até mesmo após tosse. Exames como broncoscopia, arteriografia e angiotomografia computadorizada com reconstrução 3D (TC) podem ajudar no diagnóstico de FTABCT. No entanto, em algumas situações, podem ser inconclusivos devido ao seu tamponamento por coágulos e estruturas adjacentes que mascaram o sangramento 2 . 

 O controle do sangramento é feito, na maioria das vezes, por medidas clínicas como reposição volêmica, suspensão de anticoagulantes e antiagregantes plaquetários e transfusão de hemoderivados. Além disso, também pode ser feito o tamponamento da FTABCT com o posicionamento da cânula e insuflação do balonete 1 . Nos casos mais graves, está indicada exclusão do ramo responsável pela hemorragia, por correção cirúrgica convencional ou tratamento endovascular 3 . 

 Este estudo relata o caso de uma paciente com FTABCT tratada pela técnica endovascular para controle de hemorragia maciça grave. Especial atenção é dada para sua apresentação clínica, diagnóstico e tratamento. 

## DESCRIÇÃO DO CASO

 Paciente do sexo feminino, 14 anos, apresentou história prévia de traqueostomia durante internação em unidade de tratamento intensivo aos oito anos de idade por traumatismo craniencefálico. Recebeu alta hospitalar após um mês de internação e, cerca de três meses depois, evoluiu com estenose traqueal subglótica, sendo tratada com sessões ambulatoriais de dilatação por endoscopia por seis anos. 

 No quinto ano das sessões de dilatação, apresentou hemoptise de repetição sem repercussão hemodinâmica significativa, abordada inicialmente de forma conservadora. No entanto, broncoscopia e TC identificaram FTABCT, que foi atribuída como causa do sangramento. A lesão foi corrigida por procedimento cirúrgico com ligadura da FTABCT, reconstrução do tronco arterial braquiocefálico com prótese de politetraflouroetileno expandido (PTFE) e preservação das artérias carótidas e vertebrais. 

 Após essa primeira abordagem cirúrgica, apresentou boa evolução por um ano, mantendo as sessões de dilatação; no entanto, no fim desse período, apresentou recorrência da hemoptise. Nova broncoscopia e TC de região toracocervical não identificaram foco do sangramento. 

 Foi submetida, sob anestesia geral, a arteriografia seletiva do tronco arterial braquicefálico que evidenciou a FTABCT a cerca de seis cm da carina ( Figura 1 A). Nesse momento, houve destamponamento da fístula com inundação das vias aéreas inferiores, sangramento maciço pelo tubo orotraqueal, hemomediastino com compressão do ápice do pulmão direito e consequente piora da função ventilatória ( Figura 1 B). 

**Figura 1:** (A) Arteriografia seletiva do tronco braquiocefálico, com identificação do local da fístula próximo à bifurcação das artérias subclávia e carótida comum direitas; (B) Contraste em árvore brônquica a partir de arteriografia seletiva do tronco braquiocefálico.

 Em caráter de urgência, foi posicionado balão oclusor complacente (Coda®, Cook Medical, Bloomington, EUA) em segmento médio-distal do tronco arterial braquiocefálico com obstrução total do fluxo nesse vaso ( Figuras 2 2B). Após aspiração do sangue no tubo orotraqueal, a paciente apresentou melhora da saturação com estabilidade hemodinâmica. 

**Figura 2:** (A) Balão complacente posicionado em segmento médio-distal do tronco braquiocefálico; (B) Aortografia com cateter *pigtail* 5F por acesso femoral contralateral.

 Foi realizado tratamento endovascular da FTABCT com implante de stent revestido medindo 7 × 50 mm (Wallgraft®, Boston Scientific, Marlborough, EUA) no tronco braquiocefálico e na artéria subclávia direita, proximal à origem da artéria vertebral direita, excluindo a fístula e ocluindo a origem da artéria carótida comum direita, com controle total do sangramento ( Figura 3 ). 

**Figura 3:** Arteriografia final de controle após implante de stent 70 × 50 mm (Wallgraft®, Boston Scientific, Marlborough, EUA) em tronco braquiocefálico/artéria subclávia direita, proximal ao óstio da artéria vertebral direita.

 Três dias depois, em caráter eletivo, nova cirurgia foi realizada por acesso cervical para desbridamento de tecidos desvitalizados, remoção das próteses implantadas e revascularização carotidocarotídea com preservação dos vasos cervicais. No primeiro dia pós-operatório do último procedimento cirúrgico, a paciente encontrava-se extubada, em ventilação espontânea, hemodinamicamente estável e sem sinais de déficits neurológicos. Fez uso de antibióticos por três semanas e acompanhamento clínico ambulatorial durante seis meses, sem novas complicações. 

## DISCUSSÃO

 A FTABCT é uma complicação potencialmente fatal e exige diagnóstico e tratamento precoces. Ela pode ser desenvolvida após traqueostomia, laringectomia, neoplasias ou traumas penetrantes. Em 72% dos casos, ocorre até a terceira semana após a realização da traqueostomia, mas pode ocorrer até alguns anos após o procedimento cirúrgico 1 . 

 Os sinais precoces de seu desenvolvimento incluem sangramento autolimitado com resolução espontânea, chamados “sangramentos sentinela”, presentes em 35 a 50% dos casos ou como uma hemorragia maciça e pulsação da cânula traqueal 4 ^,^ 5 . Por ser uma hemorragia arterial, o sangramento ocorre em jatos intermitentes, de coloração vermelha clara e brilhante, e costuma ser mais grave que uma hemorragia venosa, por ter uma perda mais rápida de sangue. O sangramento abundante pode ocorrer de forma súbita, sem sinais de alerta 6 . 

 O trauma na parede anterior da traqueia, decorrente da espessura de cânula utilizada da traqueostomia, de seu posicionamento em contato com a parede traqueal e da pressão do balonete, pode levar a um processo de isquemia, inflamação e ulceração com necrose transmural traqueal anterior, atingindo estruturas adjacentes como o tronco braquiocefálico 6 ^,^ 7 . Além disso, a hiperextensão do pescoço e o mau posicionamento da cânula também podem causar traumas à mucosa e são fatores que podem precipitar o desenvolvimento da FTABCT 8 . 

 A abordagem inicial nesses casos é a hiperinsuflação do balonete da traqueostomia ou tubo endotraqueal, após proteção da via aérea. Se a manobra falhar em interromper o sangramento, deve-se realizar compressão digital na parede traqueal anterior pela abertura 9 . A melhor estratégia terapêutica é evitar a colocação do tubo de traqueostomia distal ao quarto anel traqueal; evitar a hiperextensão do pescoço; e manter a pressão do balonete em menos de 20 mmHg, para impedir a necrose da mucosa 10 . 

 Geralmente, o tratamento das lesões arteriais traumáticas é realizado com técnicas tradicionais de revascularização. Cada vez mais, porém, as lesões vasculares podem ser corrigidas com eficiência por meio de procedimentos minimamente invasivos 11 ^,^ 12 . A principal vantagem do tratamento endovascular na FTABCT é a redução da morbimortalidade, sobretudo em pacientes mais graves 13 . As limitações do seu uso incluem dificuldades na colocação do stent por conta da região acometida, sendo necessários desvios cirúrgicos adicionais para manter fluxo sanguíneo, além de ausência de recursos em hospitais de menor porte. Alguns autores sugerem o uso de stents como tratamento temporário para estabilização do paciente, como neste caso, antes que um procedimento cirúrgico definitivo seja realizado, enquanto outros sugerem os stents como tratamento definitivo 14 . 

 Mesmo que alguns casos de FTABCT tenham obtido sucesso com o tratamento endovascular sem a necessidade de complementação cirúrgica (embolização, posicionamento de stent revestido ou oclusão temporária com balão), a maioria dos autores hesita em utilizar qualquer material sintético na proximidade de uma área infectada 14 . É de alta recomendação entre os autores a utilização de antibióticos por tempo prolongado em pacientes que passaram por tratamento endovascular. Nos casos de persistência de sepse ou outros sinais de complicações, deve-se considerar procedimento cirúrgico aberto 15 . 

 Apesar de tudo, os stents revestidos vêm se consagrando como opção de tratamento de traumas vasculares, por se tratar de procedimento minimamente invasivo, permitindo rápido controle do sangramento. Pode ser realizado como procedimento temporário com o objetivo de se evitar a exanguinação, seguido de um tratamento definitivo, como descrito neste relato, ou tratamento definitivo em casos selecionados 16 . 

## CONCLUSÃO

 O tratamento da FTABCT, na maioria dos casos, é realizado por medidas conservadoras, sem necessidade de intervenção. Nas formas mais graves e selecionadas, pode-se realizar o tratamento endovascular de forma efetiva ou como medida de controle do sangramento até a realização da cirurgia corretiva definitiva. O uso de stent revestido foi eficiente para o controle da hemorragia neste relato de caso e deve ser considerado em situações de sangramento maciço com grave repercussão hemodinâmica, em especial nas emergências, nas quais a demora de uma abordagem definitiva pode custar a vida do paciente.
